# Follicular steroidogenesis in random start protocols for oocyte cryopreservation

**DOI:** 10.1007/s10815-023-02883-z

**Published:** 2023-07-13

**Authors:** Giulia Galati, Edgardo Somigliana, Marta Ciaffaglione, Marco Reschini, Nicole Serra, Elena Sanzani, Paola Viganò, Elisa Polledri, Silvia Fustinoni, Ludovico Muzii, Francesca Filippi

**Affiliations:** 1 Department of Maternal and Child Health and Urology, Sapienza University, Viale Regina Elena, 324, 00161 Rome, Italy; 2 Infertility Unit, Fondazione IRCCS Ca’ Granda Ospedale Maggiore Policlinico, Milan, Italy; 3 Department of Clinical Sciences and Community Health, University of Milan, Milan, Italy; 4 Environmental and Industrial Toxicology Unit, Fondazione IRCCS Ca’ Granda Ospedale Maggiore Policlinico, Milan, Italy

**Keywords:** Oocyte, Fertility preservation, Random start, Steroids

## Abstract

**Purpose:**

Random start protocols are commonly used for oocyte cryopreservation in women with cancer. However, albeit generally reassuring, available evidence is still insufficient to rule out a sub-optimal cycle outcome. This study aimed to compare follicular steroidogenesis between women initiating the random start protocol in the luteal phase and those initiating in the follicular phase.

**Methods:**

Consecutive women with cancer scheduled for oocyte cryostorage were prospectively recruited. We excluded those requiring a concomitant letrozole assumption. All women received a standardized protocol with recombinant FSH and GnRH antagonists. At the time of oocyte retrieval, follicular fluids were pooled, and a sample was collected and frozen at −80 °C. All samples were assayed concomitantly after thawing by liquid chromatography-tandem mass spectrometry. The concentration of 15 different steroid hormones was determined.

**Results:**

Seventy-one women were recruited. Thirty-three initiated the ovarian stimulation in the luteal phase, while the remaining 38 initiated in the follicular phase. Baseline characteristics were generally similar. Cycle outcome did also not differ; the median (interquartile range) number of frozen mature oocytes was 9 (5–14) and 10 (5–21), respectively (*p* = 0.42). None of the 15 tested steroid hormones differed.

**Conclusions:**

The endocrine microenvironment surrounding oocytes is not markedly influenced by the phase of the menstrual cycle at the initiation of ovarian stimulation. This result further supports the validity of random start protocols.

**Supplementary Information:**

The online version contains supplementary material available at 10.1007/s10815-023-02883-z.

## Introduction

Global cancer incidence in women under 35 is estimated at around 430,000 per year, and about 73% of them currently survive [1]. However, cancer treatments can affect ovarian function and the chances of natural conception [2]. This issue is of great concern for cancer survivors, and it has therefore become mandatory to provide effective strategies for preserving fertility [3–6].

Oocyte cryopreservation is the most widely offered technique, but it requires ovarian stimulation [3]. Conventional protocols start on days 2–3 of the cycle to synchronize the stimulation with the natural follicular cycle, and usually last about two  weeks. Therefore, if the spontaneous onset of menstruation is awaited, these protocols can delay the beginning of cancer therapy up to 6 weeks. To reduce the duration of ovarian stimulation, “random start” protocols were introduced and are gaining consent worldwide [7].

The theoretical basis for random start stimulation originates from new insights into the physiology of folliculogenesis. The original single recruitment theory suggested that, from a cohort of 4–6-mm follicles, a single dominant follicle is selected for ovulation exclusively during the mid-follicular phase. This theory is the basis of the current practice of conventional ovarian stimulation [8]. In contrast, the recent continuous recruitment theory states that small antral follicles of 4–6 mm grow and regress continuously. The dominant follicle destined for ovulation randomly arises from this pool of antral follicles driven by endogenous gonadotropins [9]. For endocrine reasons (regression of the corpus luteum and secondary rise in FSH), this typically occurs at the beginning of the cycle, but it can theoretically occur at any time [10]. This recruitment presumably occurs continuously during the menstrual cycle even if, in most cases, it tends to assume a wave pattern: 68% of healthy women exhibit two waves of follicle development during the one interovulatory interval, and 32% exhibit three waves [11]. Based on this theory of continuous recruitment or “follicular waves,” ovarian stimulation could start at any time during the ovarian cycle [7].

Several studies supported the efficacy and feasibility of random start protocols. While the total dose of gonadotropins administered may be slightly higher with “random start” ovarian stimulation, the number of oocytes retrieved is similar [12]. A recent systematic review, including nine comparative studies, did not document significant differences between women undergoing a random start protocol and those treated with a conventional stimulation regimen initiated in the early follicular phase. The number of mature oocytes retrieved was similar (weighted mean differences +0.40 oocytes, 95% CI: −0.84/+1.66) [12]. However, none of these studies was randomized, and therefore, the quality of the evidence is not high. Moreover, and most importantly, the number of mature oocytes should be considered a surrogate measurement, the optimal outcome being the probability of having a child with those oocytes. Data on the chance of live birth can only be obtained from large case series and long follow-ups. However, this evidence is not yet available because the number of women who have thawed the stored oocytes is still modest [13].

Overall, despite the availability of reassuring evidence, investigating more in-depth the effectiveness of random start protocols remains a priority. The present study aimed to provide additional evidence on the validity of this approach by exploring the quality of ovarian steroidogenesis. Even if ovarian sex steroid production should be viewed as a surrogate modality of investigating oocyte quality, the demonstration of an unperturbed endocrine microenvironment in random start protocols would further support the validity of this approach. Increasing confidence on the effectiveness of this approach would also be important for counseling and patients’ reassurance. The primary outcome of the study was comparing levels of steroids in the follicular fluid between women initiating the random start protocol in the luteal phase and those initiating in the follicular phase (considered controls since equivalent to the conventional protocols).

## Material and methods

The present study is a single-center, biological, non-pharmacological, no-profit study, conducted at the Infertility Unit of Fondazione IRCCS Ca’ Granda Ospedale Maggiore Policlinico, Milan, Italy. The aim of the study is to evaluate the concentration of 15 steroid hormones in the follicular fluids of women with cancer undergoing oocyte cryopreservation. The recruitment period lasted from January 2018 to December 2019. The local Institutional Review Board (Comitato Etico Area B, Milano) approved our experimental protocol. All recruited women signed an informed consent form to participate.

We included women who underwent oocyte cryopreservation, had regular menstrual cycles and were diagnosed with malignant tumors. Exclusion criteria were (1) previous chemo- or radiotherapy; (2) cycle cancelation stimulation before oocyte retrieval; (3) no previous sexual intercourses because in these cases ovarian and endometrium monitoring was done using transabdominal ultrasound, a technique with insufficient accuracy for our study; (4) unclear phase of the cycle; and (5) women with hormone receptor–positive breast or ovarian cancers treated with a regimen of ovarian stimulation that included letrozole (women with hormone receptor–negative breast or ovarian cancers did not receive letrozole in our service and could be included). This latter criterion was decided because of the important effects of letrozole on steroidogenesis [14]. Women fulfilling these selection criteria were invited to participate on the day of oocyte retrieval. To note, participation in the study did not have any effect on women’s management and did not expose them to any risk. The follicular fluid is a surplus material generally discarded in assisted reproductive technology (ART) units. Women were included only once. For those performing more than one cycle, only the first one was considered.

Women were treated according to a random start protocol, which implies the possibility of starting ovarian stimulation in each of the phases of the menstrual cycle (early follicular, late follicular, or luteal) without waiting for the occurrence of menses. All women underwent transvaginal ultrasound for the assessment of antral follicle count (AFC) prior to initiating the cycle. The presence of a dominant follicle (mean diameter ≥ 11 mm) or a corpus luteum was systematically recorded. A diagnosis of corpus luteum was made in the presence of a unilocular cyst, less than 3 cm in diameter, and with diffusely thick-walled and prominent peripheral blood flow (“ring of fire” on Doppler) [15]. Assessment of the menstrual phase was made combining information on the day of the last menstruation and those obtained with ultrasound. Women in the second part of the cycle and displaying a corpus luteum were considered in the luteal phase, while those in the first part of the cycle and without a corpus luteum were considered in the follicular phase. Early follicular phase was defined based on the last menses (5 or less days before). Women were excluded if the anamnestic information on the last menses and the ultrasound assessment were not concordant (unclear phase of the cycle). Peripheral progesterone was not tested to ascertain the phase of the cycle.

The regimen of stimulation is described in detail elsewhere [16]. Briefly, all women started treatment on the day of referral, irrespective of their menstrual cycle date, with long-acting recombinant FSH 100 or 150 mcg according to body weight (< or ≥ 60 kg) (Elonva®, Merck Sharp & Dohme, UK) followed by recombinant FSH daily (Gonal-F®, Merck Serono, Italy), if needed. The use of *long-acting recombinant FSH* was shown to be non-inferior to daily recombinant FSH in this setting [16]. Women were monitored with serial transvaginal ultrasounds and, if required, with serum estrogen assessment. They were added daily with GnRH antagonists (Fyremadel® 0.25 mg, Ferring, Switzerland) when the leading follicle reached the diameter of 13–14 mm up to the time of ovulation trigger. Final oocyte maturation was triggered with GnRH agonists (Fertipeptil® 0.2 mg, Ferring, Switzerland) when at least three follicles had a mean diameter of ≥ 18 mm. Human chorionic gonadotropin (hCG) for triggering was not used. Cycles were canceled when less than three follicles developed because the balance between risks and benefits was deemed unfavorable in these circumstances. To note, the risks of oocyte retrieval (hemorrhage or infection) may have more relevance in this oncological setting because subjects are more vulnerable, in particular those with hematological disorders. Moreover, managing complications in these women may lead to significantly postpone the initiation of oncological treatments. Oocytes were collected under transvaginal ultrasound guidance 36 h after GnRH agonist injection. Oocyte denudation, maturation check, and oocyte cryopreservation were performed 2 h after the retrieval.

**Fig. 1:**
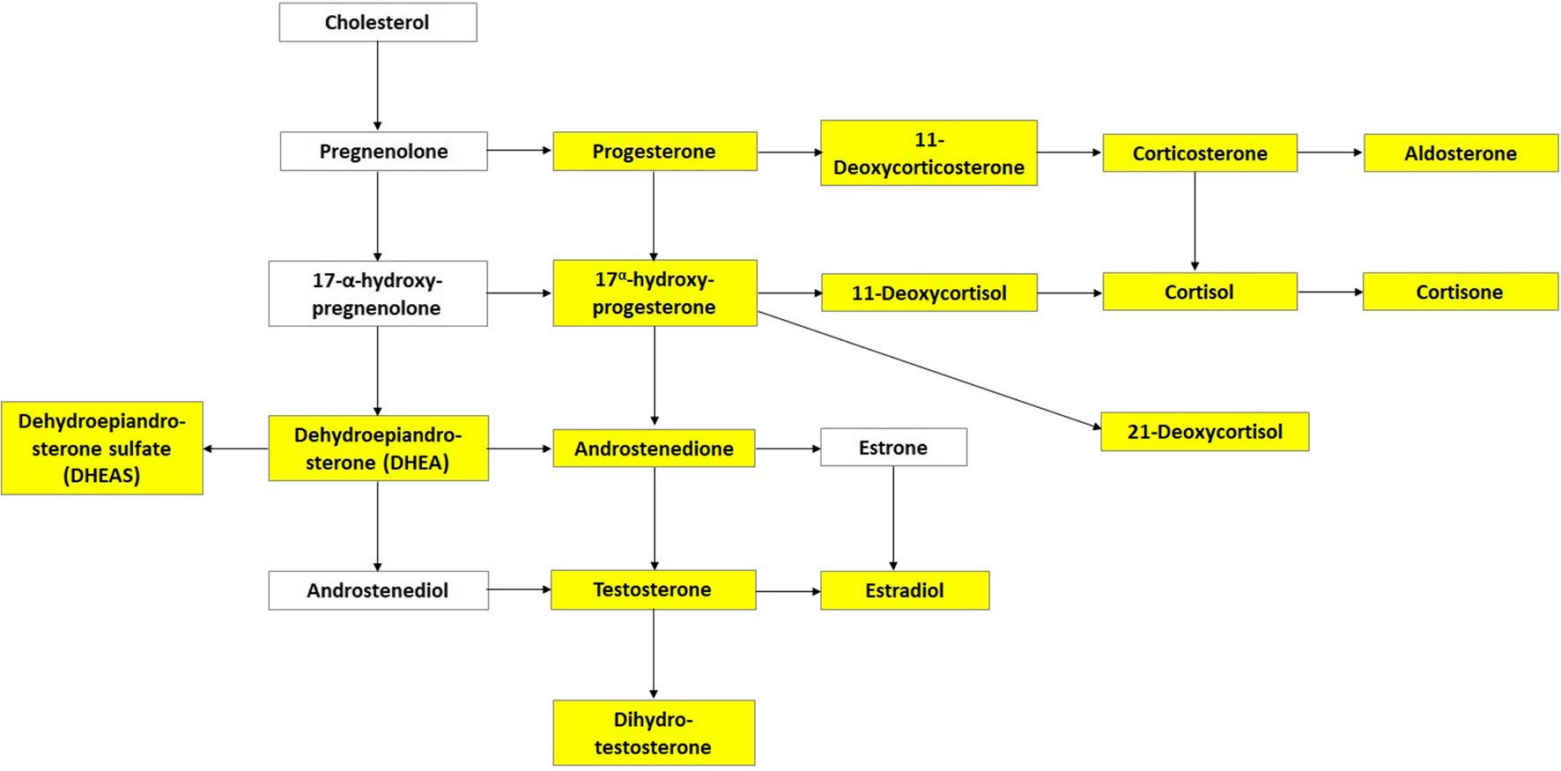
Steroidogenic cascade. Hormones evaluated in this study are highlighted in yellow. They were the following: 11-deoxycorticosterone, 11-deoxycortisol, 17-OH-progesterone, 21-deoxycortisol, aldosterone, androstenedione, corticosterone, cortisol, cortisone, dehydroepiandrosterone (DHEA), dehydroepiandrosterone sulfate (DHEAS), dihydrotestosterone, estradiol, progesterone, and testosterone

Laboratory analyses were performed on the scrap follicular fluid aspirated during oocyte retrieval. For every enrolled woman, the pool of follicular fluid was collected and then centrifugated at 2000 RPM for 10 min in 50-mL Falcon tubes at room temperature. The supernatant was collected, aliquoted in 1.5-mL Eppendorf, and cryopreserved at −20 °C. All samples were thawed concomitantly. Hormones were assessed by liquid chromatography-tandem mass spectrometry (LC-MS/MS). The levels of 15 steroids (11-deoxycorticosterone, 11-deoxycortisol, 17-OH-progesterone, 21-deoxycortisol, aldosterone, androstenedione, corticosterone, cortisol, cortisone, dehydroepiandrosterone (DHEA), dehydroepiandrosterone sulfate (DHEAS), dihydrotestosterone, estradiol, progesterone, testosterone) were determined (Fig. 1). These hormones were chosen because they comprehensively reflect the hormonal activity of the ovary [17, 18]. Only four out of 19 hormones of the cascade were not tested in our method. These four products were not included because three were intermediate substances of the chains (pregnenolone, 17-α-hydroxypregnenolone, androstenediol) and one was of scant clinical relevance in reproductive age (estrone). The procedure is explained in detail elsewhere [14, 17]. Briefly, an IVD-MS steroid kit (MassChrom, Steroids in Serum/Plasma, Chromsystems, Gräfelfing/Munich, Germany) was used. Samples were prepared according to the manufacturer’s instructions: 500 μL of each follicular fluid sample, calibrators, or quality control was placed in a solid phase extraction sample plate, previously equilibrated, with 50 μL of a deuterated internal standard mix solution and 450 μL extraction buffer. This mixed sample was then vortexed and centrifuged for 1 min at 400 × g. The supernatant of the sample was evaporated under nitrogen to dryness, reconstituted with 100 μL of reconstitution buffer, and two 40 μL aliquots were injected into the high-performance liquid chromatography system equipped with an analytical column (operating at 32 °C) for peak separation. Mobile phases were used for steroid elution. A blank calibrator matrix and six multilevel serum calibrators provided with the kit were used for calibration, and three certified quality controls of serum were used to assess within- and between-run precision and accuracy. Analysis of duplicates was performed on 10% of the samples. Relative standard deviation was up to 10%. Accuracy, evaluated for low-, intermediate-, and high-quality control, was in the range of 90–110%.

Data collected were transferred to the Statistical Package for Social Science (SPSS 26.0, IL, USA) database for subsequent analyses. Differences between the two study groups were tested using Student’s *t*-test for continuous and normally distributed variables, the Mann-Whitney test for continuous but non-normally distributed variables, and the chi-square test or Fisher exact test for categorical variables (the Fisher exact test was used if the number of subjects per category was below 5). Given the non-normal distribution of steroid hormones, these variables were compared using nonparametric statistics (Mann-Whitney test) and reported as median (interquartile range (IQR)). Two preplanned subgroup analyses were decided: (1) repeating the analyses after excluding women who were taking the combined estrogen-progesterone pills at the time of recruitment, and (2) intragroup comparison among women in the follicular phase, i.e., by comparing those initiating in the early (up to day 5) and in the late (after day 5) phases. Sample size was calculated stating as clinically relevant a fourfold increase in the frequency of cases with steroids above the 90th centile (or below the 10th centile) of the controls in women initiating stimulation in the luteal phase. In other words, a statistically significant difference between the groups is expected if 40% of women in the luteal phase displayed concentrations above the 90th centile of the distribution observed in the proliferative specimens. Setting type I and II errors at the conventional 0.05 and 0.20, this corresponded to about 66 women (33 per group).

## Results

We enrolled seventy-one women. Thirty-eight (54%) initiated the stimulation in the follicular phase, whereas the remaining thirty-three (46%) started the stimulation in the luteal phase.

Baseline characteristics of the two groups are shown in Table 1. Anamnestic data (age, body mass index (BMI), previous deliveries, seeking or not pregnancy at the time of the diagnosis), ovarian reserve variables (antral follicle count (AFC) and anti-Müllerian hormone (AMH)), and indications to cryostorage were similar. Conversely, the number of women taking birth control pills at the time of recruitment differed. This difference was expected because, in most cases, women assumed the combined estrogen-progesterone pills discontinued their use to initiate the ovarian stimulation.

**Table 1:** Baseline clinical characteristics of the study groups

Characteristics	Follicular phase	Luteal phase	*p*
	*n* = 38	*n* = 33	
Age (years)	29 (25–33)	31 (22–36)	0.70
BMI (kg/m^2^)	22.0 (19.3–23.5)	21.3 (19.9–23.4)	0.97
Previous deliveries	5 (13%)	3 (9%)	0.72
Seeking pregnancy at the time of the diagnosis	2 (5%)	2 (6%)	1.00
Combined estrogen-progesterone pills use at the time of recruitment^a^	8 (21%)	1 (3%)	0.03
Serum AMH (ng/mL)	1.84 (1.97–3.37)	2.20 (1.13–3.08)	0.74
Total AFC	18 (12–26)	17 (12–23)	0.36
Indication to oocyte cryopreservation			0.49
Hematological cancers	21 (55%)	14 (43%)	
Breast cancer	8 (21%)	7 (21%)	
Ovarian tumors	4 (11%)	3 (9%)	
Others	5 (13%)	9 (27%)	

*AFC* antral follicle count, *AMH* anti-Müllerian hormone

Data are reported as median (interquartile range) or number (percentage)

^a^These women discontinued the estroprogestin and started the stimulation on days 2–3 of the cycle (only one started in the luteal phase because of an impediment that did not allow her to initiate immediately)

Ovarian stimulation outcome is shown in Table 2. The total dose of gonadotropins, duration of stimulation, number of developed follicles, and number of oocytes retrieved did not differ.

**Table 2:** Cycle outcome in the study groups

Characteristics	Follicular phase	Luteal phase	*p*
	*n* = 38	*n* = 33	
Total dose of gonadotropins (IU)^a^	900 (600–1200)	900 (563–1350)	0.64
Duration of stimulation (days)	11 (9–11)	11 (10–12)	0.30
No. of developed follicles (diameter ≥ 11 mm)	19 (14–29)	18 (12–23)	0.24
No. of developed follicles (diameter ≥ 16 mm)	11 (6–15)	10 (5–12)	0.37
Serum estradiol on the day of trigger (pg/mL)^b^	1758 (1132–2990)	982 (672–1639)	0.05
No. of oocytes retrieved	12 (7–24)	12 (5–17)	0.31
No. of mature oocytes retrieved (frozen)	10 (5–21)	9 (5–14)	0.42

Data are reported as median (interquartile range) or number (percentage)

^a^Corifollitropin 150 mcg was set equivalent to 1400 IU of recombinant FSH and included in the calculation

^b^Data is missing in 16 and 17 cases, respectively

Follicular fluid levels of the 15 tested steroids are depicted in Table 3. No statistically significant difference was found between the two groups for any of them.

**Table 3:** Follicular steroid hormones according to the phase of the cycle at initiation of stimulation

Steroid hormones	Follicular phase	Luteal phase	*p*
	*n* = 38	*n* = 33	
Progesterone (μg/L)	421 (336–513)	477 (334–665)	0.31
11-Deoxycorticosterone (μg/L)	26.0 (21.6–31.4)	29.1 (21.9–38.5)	0.19
Corticosterone (μg/L)	2.40 (1.50–3.61)	2.44 (1.80–3.19)	0.84
Aldosterone (μg/L)	0.06 (0.02–0.09)	0.04 (0.02–0.09)	0.80
17-Hydroxyprogesterone (μg/L)	390 (305–592)	437 (374–584)	
21-Deoxycortisol (μg/L)	0.11 (0.06–0.19)	0.16 (0.07–0.23)	0.36
11-Deoxycortisol (μg/L)	0.77 (0.58–1.23)	0.78 (0.58–1.08)	0.73
Cortisol (μg/L)	55.5 (35.1–80.3)	48.0 (41.8–67.8)	0.53
Cortisone (μg/L)	12.8 (9.4–17.4)	12.3 (10.7–15.9)	0.73
DHEA (μg/L)	8.1 (5.6–13.5)	7.8 (5.1–11.6)	0.54
DHEAS (μg/L)	1314 (912–1712)	1279 (974–1724)	0.76
Androstenedione (μg/L)	4.76 (1.99–17.5)	2.64 (1.60–5.62)	0.07
Testosterone (μg/L)	0.10 (0.07–0.46)	0.07 (0.03–0.21)	0.12
DHT (μg/L)	0.12 (0.03–0.17)	0.09 (0.03–0.23)	0.84
Estradiol (μg/L)	259 (155–410)	194.0 (124.5–342.8)	0.28

*DHEA* dehydroepiandrosterone, *DHEAS* dehydroepiandrosterone sulfate, *DHT* dihydrotestosterone

Steroids were grouped based on the main branches of the cascade (see Fig. 1)

Two subgroup secondary analyses were performed to rule out confounders. First, we excluded women who were taking the combined estrogen-progesterone pills at the time of recruitment (we included 31 women in the follicular phase and 32 in the luteal phase for this analysis). Levels of steroids in the follicular fluids were mostly similar. We observed a significant difference only for androstenedione, levels being higher for women initiating stimulation in the follicular phase (Supplemental Table 1). Second, we compared women in the early and late luteal phase (26 and 12 cases, respectively). Levels of steroids in the follicular fluids did not differ except for cortisone, the concentration being higher in the early follicular phase (Supplemental Table 2).

## Discussion

The present study evaluated the quality of ovarian response in random start protocols for oocyte cryopreservation. To this aim, we compared women initiating ovarian stimulation in the luteal phase versus those initiating in the follicular phase (corresponding to the conventional modality of stimulation). The main assumption of the study was that the follicular environment could reflect the quality of the folliculogenesis. The primary outcome was the intraovarian hormonal levels, as assessed by the analysis of 15 steroids in follicular fluid recovered at the time of oocyte retrieval.

The results of the study support the validity of random start protocol since none of the tested hormones differed. A first secondary analysis excluding women taking the combined estrogen-progesterone pills (a possible confounder) did also not show main differences except for androstenedione that was higher among those initiating in the follicular phase. Androgens are claimed to increase conception rates by positively affecting follicular response to gonadotrophin stimulation [19] and could be viewed as generally beneficial. This finding may argue against the validity of stimulation initiating in the luteal phase. However, a type I error is an alternative explanation that cannot be ruled out. It is in fact plausible because of the high number of comparisons made (*n* = 15), the borderline significance (*p* = 0.03), the type of analysis (secondary), and the lack of a biological rationale for this finding. Finally, even if we generally assumed that initiating in the follicular phase could be viewed as a conventional treatment (control group), this is not entirely true since ovarian stimulation is generally started in the early follicular phase. For this reason, we did a second secondary analysis to compare initiation in the early follicular phase and in the late follicular phase. We also failed to show main differences for this comparison, except cortisone. For the same reasons illustrated in the first secondary analysis, we interpreted this single difference as a type I error. To note, in contrast to androstenedione, a role of cortisone in the quality of folliculogenesis is unlikely.

Overall, our results are in line with those that emerged from a recent systematic review including comparative studies investigating the effectiveness of random start protocols versus conventional ovarian stimulation [12]. This meta-analysis documented a slightly higher need for gonadotropins (2688 ± 660 versus 2576 ± 801 IU, *p* = 0.002), but similar numbers of mature oocytes retrieved (13.2 ± 3.7 versus 12.6 ± 4.0, *p* = NS). Recently, our group also provided evidence on the validity of random start protocols by comparing ovarian response between the ovary with the functional cyst (dominant follicle or corpus luteum) and the contralateral resting ovary of the same subjects. The results did not show any negative effect on the number of developed follicles and the number of mature oocytes retrieved per ovary [20].

Overall, evidence on the validity of random start protocols is reassuring. However, all outcomes investigated in these studies are surrogate markers of quality. The most relevant outcome remains the chance of live birth with the use of the stored oocytes. Unfortunately, evidence on the chances of success with the use of eggs stored at the time of cancer diagnosis is scant. A recent systematic review showed that most studies are poorly informative case reports [13]. Only three series including 11 [21], 49 [22], and 80 [23] women were identified. The odds of live birth were 15%, 29%, and 31%, respectively. However, the small sample size of these series hampers robust conclusions. It should also be noted that data for women treated according to traditional protocols and those treated according to random start protocols were not presented separately.

Although indirect, some reassuring results emerged also from non-oncological but similar contexts, such as observations in dual stimulation cycles (DuoStim, i.e., cycles in which a second stimulation is initiated immediately after the first oocyte retrieval and therefore in the luteal phase). Recent evidence showed that the number and quality of blastocysts obtained from cohorts of oocytes retrieved from the two sequential stimulations were similar [24]. Moreover, the same group reported reassuring data on the rate of live births. In fact, in a prospective multicenter study, no statistically significant difference was observed between euploid blastocysts derived from follicular phase and luteal phase stimulations; the live birth rates were 44% (*n* = 80/182, 95% CI: 37–51%) and 49% (*n* = 102/207, 95% CI: 42–56%; *p* = 0.30), respectively [25].

Some strengths of our study merit to be underlined. This is the first study evaluating in-depth the intraovarian hormonal environment during random start stimulation protocols. To our knowledge, only one previous study, published in 2022, provided indirect insights into the intraovarian environment during random start protocols [26]. The methodology however differed, since these authors evaluated the expression of enzymes involved in cholesterol utilization and steroid hormone biosynthesis pathways, the presence of gonadotropin receptors, and, for hormonal production, only estradiol and progesterone levels. Results were in line with our findings since the authors did not find any difference [26]. The second strength of this study is the innovativeness of the method assessing hormone levels in follicular fluid, i.e., mass spectrometry. This methodology of steroid measurement is more reliable and repeatable than that commonly used in clinical practice, a technique that has more than 30% variability [17].

On the other hand, some limitations of our study should also be recognized. First, our study is not randomized, and our results are inevitably exposed to possible confounders. However, it is difficult and ethically debatable to conduct randomized controlled trials in the urgent context of fertility preservation for cancer [16]. A possible alternative would be to investigate this issue in non-oncological settings. Second, assessment of intraovarian steroidogenesis is an indirect evidence of oocyte quality. Indeed, it provides information about the environment surrounding the oocytes, not the oocytes themselves. However, a similar hormonal production in the two phases of the ovarian cycle should be considered an additional, albeit indirect, evidence to consider in the international debate on the validity of random start protocols. Third, four of the 19 hormones constituting the whole sex steroid cascade were not tested (Fig. 1). The role of these hormones is however not crucial, being intermediate products (pregnenolone, 17-α-hydroxypregnenolone, and androstenediol) or hormones of secondary importance in young women (estrone) [17]. Finally, our sample size is relatively small, and this may affect the precision of the results. However, the sample size calculation was set up for comparison of biochemical data, not clinical data, and we achieved the planned sample size.

In conclusion, there is cumulative and consistent evidence supporting the validity of random start protocols. Our findings should generally be viewed as an additional element in favor of these regimens. However, the observation of a higher concentration of androgens in the follicular phase when excluding women taking the combined estrogen-progesterone pills is a possible concern. Further long-term clinical studies on the chances of live birth are needed for a definite conclusion.

## Supplementary information

ESM 1 (DOCX 16 kb)

ESM 2 (DOCX 15 kb)

## References

[1] Sung H, Ferlay J, Siegel RL, Laversanne M, Soerjomataram I, Jemal A (2021). Global Cancer Statistics 2020: GLOBOCAN estimates of incidence and mortality worldwide for 36 cancers in 185 countries. CA Cancer J Clin..

[2] Di Tucci C, Galati G, Mattei G, Chinè A, Fracassi A, Muzii L (2022). Fertility after cancer: risks and successes. Cancers (Basel)..

[3] Oktay K, Harvey BE, Partridge AH, Quinn GP, Reinecke J, Taylor HS (2018). Fertility preservation in patients with cancer: ASCO clinical practice guideline update. J Clin Oncol..

[4] Practice Committee of the American Society for Reproductive Medicine. Electronic address: asrm@asrm.org. Fertility preservation in patients undergoing gonadotoxic therapy or gonadectomy: a committee opinion. Fertil Steril. 2019;112:1022-1033. 10.1016/j.fertnstert.2019.09.013 31843073

[5] Anderson RA, Amant F, Braat D, D’Angelo A, de Sousa C, Lopes SM, Demeestere I, Dwek S, Frith L, Lambertini M, Maslin C, Moura-Ramos M, Nogueira D, Rodriguez-Wallberg K, Vermeulen N, ESHRE Guideline Group on Female Fertility Preservation (2020). ESHRE guideline: female fertility preservation. Hum Reprod Open..

[6] Lambertini M, Peccatori FA, Demeestere I, Amant F, Wyns C, Stukenborg JB (2020). ESMO Guidelines Committee. Fertility preservation and post-treatment pregnancies in post-pubertal cancer patients: ESMO clinical practice guidelines. Ann Oncol..

[7] Cakmak H, Rosen MP (2015). Random-start ovarian stimulation in patients with cancer. Curr Opin Obstet Gynecol..

[8] Macklon NS, Stouffer RL, Giudice LC, Fauser BC (2006). The science behind 25 years of ovarian stimulation for in vitro fertilization. Endocr Rev..

[9] De Mello Bianchi PH, Serafini P, Monteiro da Rocha A, Assad Hassun P, Alves da Motta EL, Sampaio Baruselli P, Chada BE (2010). Review: follicular waves in the human ovary: a new physiological paradigm for novel ovarian stimulation protocols. Reprod Sci..

[10] Baerwald AR, Adams GP, Pierson RA (2012). Ovarian antral folliculogenesis during the human menstrual cycle: a review. Hum. Reprod..

[11] Kirillova A, Martazanova B, Mishieva N, Semenova M (2021). Follicular waves in ontogenesis and female fertility. Biosystems..

[12] Alexander VM, Martin CE, Schelble AP, Laufer AB, Hardi A, McKenzie LJ (2021). Ovarian stimulation for fertility preservation in women with cancer: a systematic review and meta-analysis comparing random and conventional starts. J Gynecol Obstet Hum Reprod..

[13] Cobo A, García-Velasco JA, Remohí J, Pellicer A (2021). Oocyte vitrification for fertility preservation for both medical and nonmedical reasons. Fertil Steril..

[14] Dallagiovanna C, Reschini M, Polledri E, Pinna M, Ciaffaglione M, Cuce V, Somigliana E, Fustinoni S, Filippi F (2022). Effect of letrozole on follicular fluid steroids concentrations in cancer patients undergoing oocyte cryopreservation. J Assist Reprod Genet..

[15] Bonde AA, Korngold EK, Foster BR, Fung AW, Sohaey R, Pettersson DR, Guimaraes AR, Coakley FV (2016). Radiological appearances of corpus luteum cysts and their imaging mimics. Abdom Radiol (NY)..

[16] Sarais V, Paffoni A, Pagliardini L, Filippi F, Martinelli F, Mangili G, Candiani M, Papaleo E (2017). Long-acting recombinant follicle-stimulating hormone in random-start ovarian stimulation protocols for fertility preservation in women with cancer. Acta Obstet Gynecol Scand..

[17] Grassi G, Polledri E, Fustinoni S, Chiodini I, Ceriotti F, D’Agostino S, Filippi F, Somigliana E, Mantovani G, Arosio M, Morelli V (2020). Hyperandrogenism by liquid chromatography tandem mass spectrometry in PCOS: focus on testosterone and androstenedione. J Clin Med..

[18] Keevil B (2019). Steroid mass spectrometry for the diagnosis of PCOS. Medical Sciences..

[19] Nagels HE, Rishworth JR, Siristatidis CS, Kroon B (2015). Androgens (dehydroepiandrosterone or testosterone) for women undergoing assisted reproduction. Cochrane Database Syst Rev..

[20] Galati G, Serra N, Ciaffaglione M, Pinna M, Reschini M, Pisaturo V, Somigliana E, Muzii L, Filippi F (2022). Folliculogenesis in random start protocols for oocytes cryopreservation: quantitative and qualitative aspects. Reprod Sci..

[21] Specchia C, Baggiani A, Immediata V, Ronchetti C, Cesana A, Smeraldi A (2019). Oocyte cryopreservation in oncological patients: eighteen years experience of a tertiary care referral center. Front Endocrinol (Lausanne)..

[22] Diaz-Garcia C, Domingo J, Garcia-Velasco JA, Herraiz S, Mirabet V, Iniesta I (2018). Oocyte vitrification versus ovarian cortex transplantation in fertility preservation for adult women undergoing gonadotoxic treatments: a prospective cohort study. Fertil Steril..

[23] Cobo A, García-Velasco J, Domingo J, Pellicer A, Remohí J (2018). Elective and onco-fertility preservation: factors related to IVF outcomes. Hum Reprod..

[24] Cimadomo D, Vaiarelli A, Colamaria S, Trabucco E, Alviggi C, Venturella R (2018). Luteal phase anovulatory follicles result in the production of competent oocytes: intra-patient paired case-control study comparing follicular versus luteal phase stimulations in the same ovarian cycle. Hum Reprod..

[25] Vaiarelli A, Cimadomo D, Alviggi E, Sansone A, Trabucco E, Dusi L, Buffo L, Barnocchi N, Fiorini F, Colamaria S (2020). The euploid blastocysts obtained after luteal phase stimulation show the same clinical, obstetric and perinatal outcomes as follicular phase stimulation-derived ones: a multicenter study. Hum Reprod..

[26] Esmaeilian Y, Hela F, Bildik G, Akin N, İltumur E, Yusufoglu S, Yildiz CS, Keles İ, Vatansever D, Taskiran C, Yakin K, Oktem O (2023). IVF characteristics and the molecular luteal features of random start IVF cycles are not different from conventional cycles in cancer patients. Hum Reprod..

