# Supplementary material for: Follicular steroidogenesis in random start protocols for oocyte cryopreservation
Source: J Assist Reprod Genet. 2023 Jul 13;40(9):2149–56. doi: 10.1007/s10815-023-02883-z (PMC10440306; doi:10.1007/s10815-023-02883-z)
Supplement: Supplementary file 1 — (DOCX 16 kb) [file 10815_2023_2883_MOESM1_ESM.docx]

| **Supplemental Table 1.** Follicular steroids hormones according to the phase of the cycle at initiation of stimulation (excluding women taking the combined estrogen progesterone pill)**.** | | | |
| --- | --- | --- | --- |
|  |  |  |  |
| Steroid hormones | Follicular phase | Luteal phase | p |
|  | n=30 | n=32 |  |
|  |  |  |  |
| Progesterone (μg/l) | 423 [361 - 513] | 473 [333 - 670] | 0.25 |
| 11-deoxycorticosterone (μg/l) | 26.4 [22.3 - 31.4] | 29.2 [22.7 - 39.3] | 0.17 |
| Corticosterone (μg/l) | 2.70 [2.07 - 3.65] | 2.49 [1.80 - 3.24] | 0.39 |
| Aldosterone (µg/l) | 0.06 [0.02 - 0.09] | 0.04 [0.02 - 0.09] | 0.94 |
|  |  |  |  |
| 17-hydroxyprogesterone (μg/l) | 401 [311 - 640] |  | 0.52 |
| 21-deoxycortisol (μg/l) | 0.11 [0.06 - 0.20] | 0.16 [0.07 - 0.23] | 0.31 |
| 11-deoxycortisol (μg/l) | 0.77 [0.56 - 1.36] | 0.78 [0.61 - 1.10] | 0.84 |
| Cortisol (μg/l) | 56.6 [39.0 - 80.6] | 48.6 [40.6 - 68.2] | 0.41 |
| Cortisone (μg/l) | 12.8 [8.3 - 15.7] | 12.4 [10.6 - 15.9] | 0.48 |
|  |  |  |  |
| DHEA (μg/l) | 8.3 [6.2 - 13.5] | 7.9 [5.0 - 11.9] | 0.36 |
| DHEAS (μg/l) | 1,340 [900 - 1,731] | 1,267 [974 - 1,645] | 0.98 |
|  |  |  |  |
| Androstenedione (μg/l) | 4.9 [2.9 - 17.5] | 2.5 [1.6 - 5.7] | 0.03 |
| Testosterone (μg/l) | 0.10 [0.07 - 0.57] | 0.06 [0.03 - 0.21] | 0.07 |
| DHT (μg/l) | 0.12 [0.03 - 0.16] | 0.09 [0.03 - 0.23] | 0.84 |
| Estradiol (μg/l) | 299 [150 - 455] | 216 [120 - 349] | 0.21 |
|  |  |  |  |
|  |  |  |  |
| DHEA: dehydroepiandrosterone. DHEAS: dehydroepiandrosterone sulfate | | |  |
| DHT: dihydrotestosterone |  |  |  |
| Steroids were grouped based on the main branches of the cascade (see figure 1). | | |  |
