# Supplementary material for: Follicular steroidogenesis in random start protocols for oocyte cryopreservation
Source: J Assist Reprod Genet. 2023 Jul 13;40(9):2149–56. doi: 10.1007/s10815-023-02883-z (PMC10440306; doi:10.1007/s10815-023-02883-z)
Supplement: Supplementary file 2 — (DOCX 15 kb) [file 10815_2023_2883_MOESM2_ESM.docx]

| **Supplemental Table 2.** Follicular steroids hormones according to the phase of the cycle at initiation of stimulation (early vs late follicular phase)**.** | | | |
| --- | --- | --- | --- |
|  |  |  |  |
| Steroid hormones | Early follicular phase | Late follicular phase | p |
|  | n=26 | n=12 |  |
|  |  |  |  |
| Progesterone (μg/l) | 423 [320 - 536] | 418 [366 - 488] | 0.96 |
| 11-deoxycorticosterone (μg/l) | 25.6 [21.4 - 31.4] | 26.9 [22.9 - 39.3] | 0.49 |
| Corticosterone (μg/l) | 2.30 [1.50 - 3.65] | 3.1 [1,6 - 3.4] | 0.61 |
| Aldosterone (µg/l) | 0.06 [0.01 - 0.09] | 0.08 [0.03 - 0.12] | 0.30 |
|  |  |  |  |
| 17-hydroxyprogesterone (μg/l) | 387 [305 - 574] | 390 [300 - 698] | 0.79 |
| 21-deoxycortisol (μg/l) | 0.11 [0.07 - 0.19] | 0.12 [0.05 - 0.23] | 0.94 |
| 11-deoxycortisol (μg/l) | 0.76 [0.64 - 1.16] | 1.11 [0.38 - 1.59] | 0.72 |
| Cortisol (μg/l) | 57.9 [38.3 - 80.8] | 53.3 [26.8 - 77.0] | 0.45 |
| Cortisone (μg/l) | 13.8 [11.0 - 18.4] | 9.2 [6.5 - 14.0] | 0.03 |
|  |  |  |  |
| DHEA (μg/l) | 7.9 [5.8 - 13.8] | 9.1 [5.2 - 13.1] | 0.82 |
| DHEAS (μg/l) | 1,314 [921 - 1,695] | 1,232 [722 - 1,803] | 0.96 |
|  |  |  |  |
| Androstenedione (μg/l) | 4.7 [2.0 - 12.6] | 5.2 [2.0 - 18.4] | 0.87 |
| Testosterone (μg/l) | 0.10 [0.07 - 0.36] | 0.10 [0.07 - 0.64] | 0.77 |
| DHT (μg/l) | 0.09 [0.03 - 0.18] | 0.14 [0.03 - 0.17] | 0.51 |
| Estradiol (μg/l) | 250 [172 - 390] | 270 [81 - 535] | 0.77 |
|  |  |  |  |
|  |  |  |  |
| DHEA: dehydroepiandrosterone. DHEAS: dehydroepiandrosterone sulfate | | |  |
| DHT: dihydrotestosterone |  |  |  |
| Steroids were grouped based on the main branches of the cascade (see figure 1). | | |  |
